# Salivary Calculus

**Published:** 1863-09

**Authors:** John C. Hupp

**Affiliations:** Wheeling, West Va.


Salivary Calculus.—*By John C. Hupp, M. D., Wheeling, West Va.*—In the month of October, 1860, I extracted the enclosed specimen of salivary calculus. It occupied an excretory duct of the sublingual gland under the right side of the tongue.

The subject, aged thirty-eight, was G. McD., of Beallsville, Monroe county, Ohio. At the time I was consulted and extracted the calculus, the tumor had existed over two years, and was the size of an ordinary almond. After subsidence of a severe inflammation of the throat, complicated with external swelling, which was followed by ulceration of the mouth and tongue, the tumor first attracted attention. The patient never suffered severe pain in the tumor, but when under the influence of cold he often experienced an "unpleasant, cramping sensation," also, occasionally, lancinating pain.

During the interval of two years referred to, he could not discover much increase in the size of the tumor, except when under the influence of cold, at which time it became inflamed and enlarged. Pressing the side of the tongue upward, the tumor constantly interfered with the free use of that organ, which inconvenience was greatly increased when the tumor was inflamed.

For about six weeks after the discovery of the tumor, ineffectual efforts were made to reduce the "morbid growth" by means of caustics, and from that time till it came under my care the use of remedies was abandoned. Patient writes me: "I often felt uneasy on account of the tumor, fearing it might be of the nature of a cancer, and I was very much surprised when, upon examining, you told me there was something confined there that should be removed. * * * I have been entirely relieved and discover no appearance of its return."—*Med. and Surg. Reporter*, Aug. 13, 1863.

